# Listeria in Pregnancy—The Forgotten Culprit

**DOI:** 10.3390/microorganisms12102102

**Published:** 2024-10-21

**Authors:** Vladimír Kraus, Beáta Čižmárová, Anna Birková

**Affiliations:** 1 Department of Gyneacology and Obstetrics, Faculty of Medicine, Pavol Jozef Šafárik University in Košice, Trieda SNP 1, 040 11 Košice, Slovakia; vladimir.kraus1@upjs.sk; 2 Department of Medical and Clinical Biochemistry, Faculty of Medicine, Pavol Jozef Šafárik University in Košice, Trieda SNP 1, 040 11 Košice, Slovakia; beata.cizmarova@upjs.sk

**Keywords:** *Listeria monocytogenes*, listeriosis, pregnancy, neonatal listeriosis, granulomatosis infantisepticum

## Abstract

*Listeria monocytogenes* is a Gram-positive bacterium that causes listeriosis, a severe foodborne illness that is particularly dangerous during pregnancy. It thrives in diverse environments, including refrigerated conditions and food production facilities, due to its adaptability to varying temperatures, pH levels, and salt concentrations. Its virulence stems from the ability to invade host cells, particularly macrophages and epithelial cells, and avoid, or at least postpone, immune detection by utilizing virulence factors such as internalins, listeriolysin O, and actin assembly-inducing protein. This intracellular motility and biofilm formation make LM a persistent pathogen in food safety and public health. Pregnant women are at a much higher risk of listeriosis, which can result in serious fetal complications such as miscarriage, stillbirth, and preterm labor due to LM’s affinity for placental tissues. The vertical transmission of LM from mother to fetus can lead to neonatal listeriosis, which can result in sepsis and meningitis, with high mortality rates if not promptly treated. Early diagnosis and treatment with antibiotics, such as ampicillin or gentamicin, are crucial for maternal and neonatal outcomes.

## 1. Introduction

*Listeria monocytogenes* (LM) is a bacterium recognized for causing listeriosis, a severe foodborne disease. Given the rarity of listeriosis and the often mild nature of its symptoms, this infection remains relatively obscure to the general public and is not typically among the initial considerations of healthcare providers when evaluating symptomatic patients. Pregnant women represent one of the most vulnerable populations, with the potential for severe consequences for both mother and the fetus. This review aims to provide a thorough overview of listeriosis, covering its diagnosis and treatment, while also delving into the mechanisms of vertical transmission and the clinical manifestations of neonatal listeriosis.

LM is a Gram-positive, rod-shaped microorganism classified within the genus Listeria. It exhibits facultative anaerobism, allowing it to thrive in both aerobic and anaerobic conditions, which contributes to its adaptability across diverse environments [1]. Structurally, LM is a small, non-spore-forming, motile rod, typically ranging in size from 0.5 to 2 μm in length and approximately 0.5 μm in diameter. Under optimal conditions, LM maintains a classic rod-shaped morphology, though environmental stressors can induce morphological variation, occasionally leading to a coccoid (spherical) form. At temperatures below 30 °C (86 °F), LM exhibits motility due to the presence of peritrichous flagella, which are distributed across its surface, producing a characteristic “tumbling” motion. However, at human body temperature (37 °C/98 °F), the flagella are not expressed, rendering the bacterium non-motile [1].

LM is regarded as a highly resilient pathogen, owing to several distinctive characteristics. One of its most notable attributes is its psychrotrophic nature, enabling it to grow at low temperatures, including refrigeration levels (0–4 °C/32–39 °F), although its optimal growth temperature lies between 30 °C and 37 °C (86–98 °F) [2]. 

**Figure 1:**
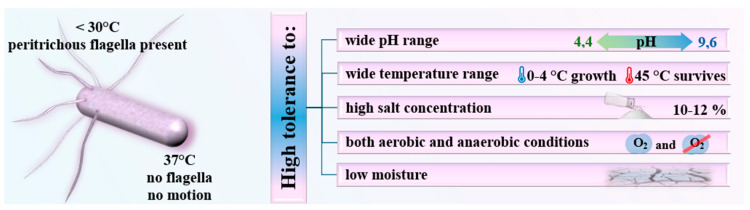
*Listeria monocytogenes* is highly resilient and can survive under a wide range of environmental conditions.

LM also demonstrates considerable adaptability to varying environmental conditions. It can survive and proliferate across a broad pH spectrum, ranging from 4.4 to 9.6. This acid tolerance allows it to endure in acidic food environments, such as fermented products. Additionally, LM is halotolerant, capable of surviving in elevated salt concentrations, typically up to 10–12%, which explains its persistence in processed foods like cured meats and cheeses [3]. Most common sources of LM infection are listed in Table 1. Moreover, as a facultative anaerobe, LM can grow in both oxygen-rich and oxygen-depleted environments, affording it the versatility to colonize a wide array of ecological niches, including soil, food products, and human tissues. These survival mechanisms contribute to its role as a formidable pathogen in food safety and public health [3]. All of its unique abilities, which make LM a guest who can easily overstay its welcome, are summarized in Figure 1.

LM is classified as an intracellular pathogen, signifying its ability to invade and replicate within host cells, particularly targeting phagocytic cells such as macrophages and also epithelial cells and neurons. To achieve intracellular survival and proliferation, LM employs a variety of virulence factors.

One key group of virulence factors are internalins (Inl, with InlA and InlB being the most known), which are surface proteins that mediate bacterial adherence and the invasion of host cells. These proteins interact with specific host–cell receptors, allowing LM to penetrate the intestinal epithelium and breach critical barriers such as the placental and blood–brain barriers. Inls are distinguished by the presence of leucine-rich repeat domains. To date, over 25 distinct Inls have been identified within LM [4,5].

Another pivotal virulence factor is listeriolysin O (LLO), a pore-forming toxin that facilitates the bacterium’s escape from the phagosome into the host–cell’s cytoplasm following engulfment. This cholesterol-dependent cytolysin disrupts the phagosomal membrane, enabling LM to evade lysosomal degradation by the host immune system and establish a replicative niche within the cytoplasm, thereby promoting intracellular infection and further dissemination [6].

The actin assembly-inducing protein (ActA) plays a critical role in the intracellular motility of LM. Upon entry into the host–cell cytoplasm, LM hijacks the host’s actin cytoskeleton to facilitate its movement through a mechanism known as actin-based motility. ActA is essential in this process as it catalyzes the polymerization of actin filaments at one pole of the bacterium, generating actin tails that propel the bacterium forward [2].

LM’s resilience in the environment is significantly enhanced by its capacity to form biofilms. Biofilms are intricate communities of microorganisms enshrouded in a self-produced extracellular matrix that adheres to various surfaces. A key factor in LM’s biofilm formation is its adhesion protein (LAP), which facilitates attachment to surfaces. This protein binds to host–cell receptors like HSP60 (Heat Shock Protein 60), a chaperonin involved in protein folding, enhancing the bacterium’s ability to adhere and initiate biofilm formation, particularly on biotic surfaces [7].

**Figure 2:**
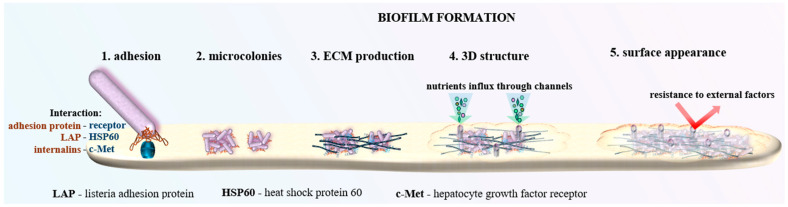
*Listeria monocytogenes* forms robust biofilms on various surfaces, providing protection against environmental stresses and sanitizing agents.

This structure not only renders the bacteria resistant to desiccation, sanitizing agents, and disinfectants, but also facilitates persistent contamination within food processing environments. Biofilms can establish themselves on equipment, drains, and other surfaces, posing a continuous threat of contamination if not properly managed [3]. Figure 2 shows a schematic illustration of LM’s biofilm.

The remodeling of cellular junctional architecture via the LAP–HSP60 interaction, facilitating the passage of LM through the epithelial barrier, remains poorly understood. A study employing the gerbil model, which is permissive to Inl A/B-mediated pathways similar to humans, revealed that LM traverses the intestinal villi as early as 48 h post-infection. LAP facilitates LM translocation through the endocytosis of the cell–cell junctional complex in enterocytes that are lacking luminal E-cadherin. In contrast, InlA mediates LM translocation in cells exhibiting apical E-cadherin during cell extrusion and mucus expulsion from goblet cells. LAP exploits caveolar endocytosis to traffic key junctional proteins to early and recycling endosomes. Pharmacological inhibition in a cell line and with the genetic knockout of caveolin-1 in mice prevents LAP-induced intestinal permeability, junctional endocytosis, and subsequent LM translocation. Moreover, LAP-HSP60-dependent tight junction remodeling is essential for InlA-mediated access to E-cadherin, allowing LM to cross the intestinal barrier in InlA-permissive hosts [8].

Moreover, LM’s pathogenicity is bolstered by its adeptness at evading the host immune response. Its capability to invade and replicate within host cells shields it from immune components that predominantly function in the extracellular milieu. Additionally, LM has evolved a sophisticated mechanism for direct cell-to-cell dissemination, allowing it to transition between host cells without exposure to the extracellular environment, further circumventing immune detection [9].

**Figure 3:**
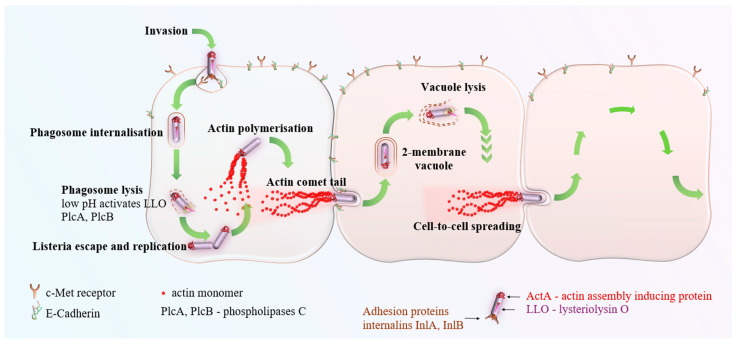
*Listeria monocytogenes* infects neighboring cells by utilizing a process called cell-to-cell spread. While a similar process is used by different intracellular pathogens, ActA is a unique tool in the arsenal of LM.

LM utilizes the host–cell’s actin cytoskeleton to facilitate its intracellular movement, a process orchestrated by the ActA protein. This interaction results in the formation of actin “comet tails”, which propel the bacterium through the cytoplasm and into cellular protrusions. These protrusions are subsequently engulfed by adjacent cells, leading to the formation of a double-membrane vacuole surrounding the bacterium. LM then employs virulence factors such as LLO and phospholipases to lyse the vacuolar membranes, thereby releasing itself into the cytoplasm of the newly infected host cell [1]. This cell-to-cell spread is visualized in Figure 3.

Once internalized, LM has the ability to delay the maturation of the phagosome, a membrane-bound compartment that typically fuses with lysosomes to degrade engulfed pathogens. This delay provides LM with a critical window of opportunity to escape into the cytoplasm before lysosomal degradation can occur. Additionally, LM secretes virulence factors, including InlC, which disrupt host–cell signaling pathways, particularly those involved in the innate immune response such as the NF-κB pathway. Blocking the translocation of NF-κB to the nucleus leads to the dampening of the host’s inflammatory response by reducing the production of pro-inflammatory cytokines, like TNF-α and IL-6. InlC also appears to suppress neutrophil recruitment [10,11]. The adaptive response to Listeria involves Th1-type CD4+ T cells, which produce IFN-γ and activate cytotoxic CD8+ T cells. These cells are critical for recognizing and killing infected host cells. However, the shift toward a Th2-dominant immune profile during pregnancy can reduce the efficiency of Th1 responses, making it more challenging to fight off intracellular pathogens like LM [12,13].

LM predominantly relies on fermentation, converting glucose into lactic acid under anaerobic conditions. However, LM exhibits metabolic flexibility by utilizing a wide range of carbohydrates and amino acids. This metabolic versatility enables the bacterium to thrive in environments with varying nutrient availability, including the nutrient-poor conditions that are often encountered in food production facilities.

The genome of LM is relatively compact, comprising approximately 2.8 million base pairs and encoding around 2800 genes. This streamlined genome reflects the bacterium’s adaptability and versatility, encompassing genes that are essential for virulence, stress response, and metabolic functions. Furthermore, the horizontal gene transfer between LM and other environmental bacteria plays a significant role in its ability to acquire resistance to adverse conditions, such as exposure to disinfectants or antibiotics [14,15,16].

## 2. Listeriosis

Listeriosis, a disease caused by LM, primarily affects vulnerable populations, including pregnant women, neonates, the elderly, and immunocompromised individuals. While infection in healthy individuals typically manifests as mild flu-like symptoms, those in high-risk groups may experience severe complications such as meningitis, septicemia, and, in the case of pregnant women, stillbirth or miscarriage. Although listeriosis is relatively rare, it carries a significantly higher mortality rate compared to other foodborne illnesses. Timely diagnosis and intervention are essential as severe cases often necessitate hospitalization and antibiotic therapy [1].

Globally, the incidence of listeriosis, encompassing both maternal and non-maternal cases, is estimated to range from 0.1 to 1.5 cases per 100,000 individuals annually. In the United States, the estimated incidence of neonatal listeriosis is approximately 3 to 6 cases per 100,000 live births, whereas in Europe, this rate is estimated at 2 to 8 cases per 100,000 live births [6].

**Figure 4:**
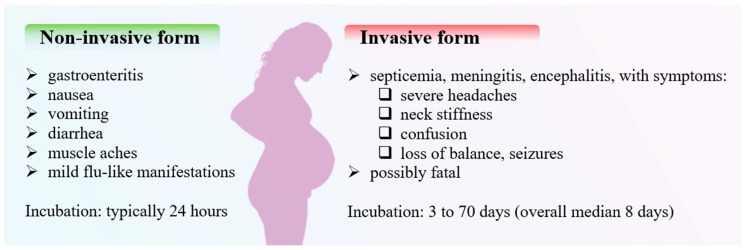
Two forms of listeriosis, with its symptoms.

Listeriosis manifests in two primary forms: non-invasive and invasive (Figure 4). Non-invasive listeriosis, the milder form, predominantly affects healthy individuals. Its symptoms include gastroenteritis, nausea, vomiting, diarrhea, muscle aches, and mild flu-like manifestations. These symptoms typically emerge within 24 h to a few days after the consumption of contaminated food and are generally self-limiting, resolving without the need for medical intervention in most cases [2].

In contrast, invasive listeriosis is considerably more severe, leading to systemic infections, particularly in high-risk populations. This form may present as meningitis, septicemia, or encephalitis, with symptoms such as severe headaches, neck stiffness, confusion, loss of balance, seizures, and, in some cases, death. The incubation period for invasive listeriosis is typically longer, with symptoms appearing between 3 and 70 days post-exposure [2,17].

Efforts to mitigate the spread of LM in food production and handling require rigorous monitoring, food recalls, and public health campaigns. Preventative measures primarily focus on proper food handling practices, such as thorough cooking, avoiding cross-contamination, and maintaining the cleanliness of food preparation surfaces [1].

The diagnosis of listeriosis is typically confirmed through the analysis of blood or cerebrospinal fluid, where LM can be cultured and identified. While the direct visualization of LM through microscopy is theoretically possible, the low bacterial load both in the blood and cerebrospinal fluid make this approach uncommon, and blood cultures remain the gold standard [18].

The bacterium usually requires approximately 36 h to grow sufficiently for detection. Early diagnosis is crucial, particularly in cases of invasive listeriosis, due to the potential for rapid disease progression. Serological tests that detect antibodies against LLO toxin may aid in diagnosing both invasive and non-invasive forms of listeriosis; however, these tests are not commonly employed in routine diagnostics. Polymerase chain reaction testing, which can swiftly detect LM DNA, including in food samples, remains experimental and is not yet widely available [2,19].

Treatment for listeriosis generally involves the administration of antibiotics, with ampicillin and gentamicin being the most frequently prescribed. The duration of therapy varies depending on the severity of the infection and the patient’s overall health. In cases where invasive listeriosis develops—particularly meningitis or sepsis—intensive care may be required, and despite prompt treatment, the condition may still result in fatal outcomes [2].

## 3. Listeria in Pregnancy

Pregnant women are significantly more susceptible to contracting listeriosis due to the suppression of cell-mediated immunity that occurs during pregnancy, which weakens the body’s ability to combat infections. Intracellular pathogens, such as LM, Toxoplasma gondii, and viruses like cytomegalovirus, rely on a robust Th1-mediated immune response for effective clearance, which includes the production of IFN-γ and the activation of macrophages and cytotoxic T cells. However, as previously mentioned, the reduced Th1 activity in pregnant women compromises this response, thereby allowing these pathogens to more easily evade host immune defenses [20,21].

Furthermore, LM exhibits a particular affinity for the placenta. As a result, pregnant women are approximately 17 times more likely to develop listeriosis compared to the general population [6]. This is why it is wise to avoid foods most commonly associated with the presence of LM (Table 2).

## 4. Maternal Listeriosis

While maternal listeriosis typically results in mild, flu-like symptoms in pregnant women, the primary concern is the pathogen’s ability to cross the placenta and infect the fetus. Maternal symptoms are generally non-specific, including fever, headache, myalgia, and gastrointestinal disturbances. It is important to note that pregnancy does not inherently increase the risk of neurolisteriosis, and central nervous system involvement in pregnant women without underlying immunodeficiencies is exceedingly rare [9].

Despite the mild nature of maternal symptoms, the fetal consequences can be severe. Maternal listeriosis in early pregnancy carries a 65% risk of miscarriage and can result in stillbirth at any stage of gestation. The likelihood of live birth increases with advancing gestational age, with studies suggesting that each additional week of gestation at the time of infection raises the odds of fetal survival by 33%. Furthermore, Listeria infection significantly heightens the risk of preterm labor and delivery [9,22]. However, there is insufficient data to determine whether early Listeria infection contributes to specific congenital defects [23].

In addition to the previously mentioned diagnostic methods, several others are particularly relevant in pregnancy. Placental cultures are considered the gold standard for diagnosing maternal-fetal listeriosis, offering greater sensitivity than maternal blood cultures (80% vs. 55%). Bacterial cultures obtained from placental biopsy samples demonstrate even higher specificity. By directly sampling placental tissue, the biopsy increases the likelihood of isolating LM due to a higher concentration of the bacterium. Furthermore, placental biopsies permit histopathological examination, which can reveal hallmark signs of Listeria infection, such as microabscesses, chorioamnionitis, and inflammatory infiltrates, even in the absence of positive cultures. The sensitivity of placental biopsy is reported to be 100%. Placental biopsies are typically indicated in cases where bacterial infection is suspected to have caused pregnancy loss or neonatal infection, particularly in instances of unexplained fetal demise in the third trimester or in preterm labor. This procedure is especially valuable when routine blood cultures fail to identify the causative pathogen. Given the associated risks to both the fetus and the mother, placental biopsies are generally conducted post-delivery rather than during pregnancy. Another diagnostic option is the culture of amniotic fluid, although its sensitivity is lower than that of placental cultures [9,24].

It is recommended to inform the laboratory of a suspected LM infection to ensure the use of appropriate culture techniques and to minimize false negatives as LM may be misidentified as other bacteria, such as diphtheroids, due to its microscopic appearance.

Certain strains of LM appear to have a greater propensity to cause adverse pregnancy outcomes. Among the thirteen identified serotypes of LM, only four (1/2a, 1/2b, 1/2c, and 4b) are commonly implicated in clinical listeriosis worldwide. Of these, serotype 4b has been disproportionately associated with pregnancy-related listeriosis, suggesting that it may possess specific characteristics that enhance its ability to infect pregnant women and/or traverse the placental barrier [9,22].

Further research has sought to clarify the relationship between specific LM strains and adverse pregnancy outcomes. Several distinct clonal complexes (CCs) within serotype 4b have been identified and associated with such outcomes. CCs are groups of closely related strains that share common genetic traits and ancestry. These complexes are categorized using molecular typing techniques such as multilocus sequence typing, which examines the sequences of several housekeeping genes to classify bacterial strains into particular lineages. Among the CCs associated with adverse pregnancy outcomes are CC1, CC2, CC4, and CC6, with CC4 demonstrating the highest tendency towards placental infection. Strains within CC4 exhibit elevated expression of the internalin InlB, a key factor in the bacterium’s ability to invade non-phagocytic cells. This increased expression of InlB likely enhances the strain’s invasiveness, contributing to a greater capacity for placental colonization and vertical transmission [5].

**Figure 5:**
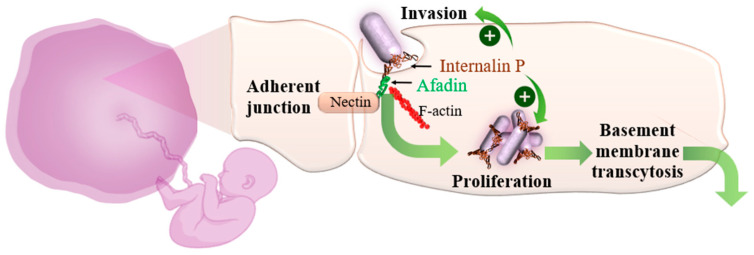
Placenta-specific affinity of LM based on internalin P–afadin complex formation.

LM demonstrates a specific affinity for the placenta, which enhances its likelihood of infecting and proliferating within placental tissues. This tropism is largely due to LM’s ability to interact with particular receptors on placental cells, thereby facilitating its entry and survival. A recently identified virulence factor, internalin P (InlP), is notably associated with placental infection. Unlike the more widely recognized internalins, namely InlA and InlB, which mediate LM invasion into a range of cell types, InlP shows a distinct preference for placental tissues [25,26]. InlP targets afadin, a protein integral to cell junctions which plays a crucial role in maintaining the structural integrity of epithelial and endothelial barriers, including those within the placenta (Figure 5). The binding of InlP to afadin is believed to aid LM in traversing the basement membrane—a thin, fibrous layer that separates the placental trophoblast cells from the underlying maternal tissues. Additionally, InlP has been shown to promote LM proliferation in human placental organ cultures and trophoblast cells, further supporting its role in enhancing placental infection [2,27,28].

However, it appears that not all trophoblast cells exhibit the same level of sensitivity to LM. Syncytiotrophoblast cells are resistant to LM adhesion on their surface, whereas cytotrophoblast cells remain susceptible [29].

Despite these findings, there is an ongoing debate as to whether LM exhibits an active or passive tropism for the placenta. The active tropism hypothesis suggests that LM has evolved specific mechanisms to deliberately target and invade placental tissue, facilitating its crossing of the placenta. Conversely, the passive tropism perspective proposes that the placenta’s unique immunological properties and the presence of susceptible cell types inherently predispose it to serve as an inadvertent reservoir for LM. It is well established that the maternal immune system within the placenta undergoes a shift from cell-mediated immunity, characterized by decreased Th1 responses, to a predominance of Th2 responses, which are associated with humoral immunity. This immunological adaptation helps prevent fetal rejection but also creates a more favorable and protective environment for LM, potentially enhancing its survival and proliferation within the placental tissues [30,31].

The epithelial cells that constitute the placental barrier serve as the initial responders to LM infection. These cells express Toll-like receptors that recognize bacterial components and initiate the production of pro-inflammatory cytokines, such as IL-6 and TNF-α. Additionally, they secrete chemokines, including IL-8 and MIP-1α/β, which function to recruit neutrophils and other immune cells to the infection site. Hofbauer cells (HBCs), which are resident macrophages in the placenta, also play a role in the innate immune response. Although these cells can phagocytose LM, they can themselves become infected, potentially contributing to the spread of the bacteria. Following LM infection, HBCs transition from an M2 (anti-inflammatory) to an M1 (pro-inflammatory) phenotype, releasing cytokines such as IL-1β and IL-18. Furthermore, decidual natural killer cells contribute to the immune response by transferring the antimicrobial peptide granulysin to infected trophoblasts, thereby directly targeting and combating the bacteria [5,27].

By 24 h post-infection, LM triggers a broad inflammatory response across all placental cell lineages. This response is characterized by the upregulation of cytokines and chemokines, including CXCL3, CXCL8, CCL20, CCL4, and CCL3, particularly in trophoblasts that form the placental barrier. The study also demonstrated protein-level upregulation and observed this response in other placental lineages, such as fibroblasts, endothelial cells, and HBCs. HBCs play a pivotal role in the placental defense against LM by upregulating specific antimicrobial and pro-inflammatory molecules. The HBCs enhance the expression of TLR2, a critical pathogen recognition receptor that activates the innate immune response. Also, HBCs upregulate several molecules with established antibacterial properties, including S100A8, SLC11A1, LYN, and HMGB1. Additionally, HBCs show increased expression of the already mentioned IL-1β. Its receptor components (IL-1R1 and IL1RAP) are predominantly expressed by fibroblasts and perivascular cells within the placenta. This suggests that IL-1β produced by HBCs primarily targets these cell types, likely contributing to a broader inflammatory response within placental tissue. Notably, HBCs also upregulate amphiregulin in response to LM, which may activate the epidermal growth factor receptor (EGFR) on trophoblasts, potentially supporting their survival and regeneration after infection [32].

These innate immune responses are frequently inadequate to eradicate the infection as LM is capable of surviving and replicating within macrophages and other cell types, thereby evading initial innate defenses. This persistence allows for the continued bacterial proliferation and extensive colonization of the placenta. Such extensive colonization ultimately provokes a robust inflammatory response, characterized by a significant influx of neutrophils and inflammatory mediators. While this intense response aims to eliminate the bacteria, it can inadvertently disrupt fetal tolerance, leading to adverse outcomes, including preterm labor, fetal distress, and, potentially, fetal demise [33,34].

In the treatment of listeriosis, β-lactam antibiotics are considered the first-line therapy. Ampicillin and amoxicillin are the preferred agents for treating listeriosis during pregnancy. These antibiotics inhibit bacterial cell wall synthesis by blocking peptidoglycan cross-linking, which is crucial for bacterial viability. High doses are recommended to ensure therapeutic efficacy, with intravenous ampicillin administered at 6–12 g/day, divided into 2 g doses, or oral amoxicillin at 100 mg/kg/day. The duration of treatment typically extends until delivery or for at least 14 days (up to 21 days, depending on the infection’s severity) to maintain adequate drug levels in the placenta and fetus. In cases of severe maternal infection, the addition of an aminoglycoside, such as gentamicin, is advised due to its synergistic bactericidal effect. However, aminoglycosides are associated with potential toxicity, particularly to the fetus, so their use is generally confined to more severe cases and limited to a maximum of 10 days. For patients with a penicillin allergy, alternative treatments include trimethoprim/sulfamethoxazole or erythromycin [2,6,35].

Erythromycin, as a second-line treatment, is administered at a dosage of 4 g per day for a duration of 14 days. The combination of trimethoprim/sulfamethoxazole may also be utilized, available in both oral and intravenous forms; however, its use during pregnancy poses potential risks, particularly due to trimethoprim’s folate antagonism, which may adversely affect fetal development, especially in the first trimester. Additionally, several classes of antibiotics, including cephalosporins, clindamycin, and chloramphenicol, are known to be ineffective against LM. Since the symptoms of listeriosis are vague and its sequelae can be devastating, clinical suspicion often leads to over-treatment [36,37]. While maternal outcomes are typically favorable with appropriate treatment, fetal prognosis is influenced by various factors, which are discussed further in the article.

## 5. Neonatal Listeriosis

LM typically transmits from the mother to the fetus through the placenta, with transmission most commonly occurring during the third trimester when the placenta is more susceptible to infection.

It is entirely plausible that listeriosis is underdiagnosed during the earlier trimesters and may not be suspected in cases of early pregnancy loss. However, reports of listeriosis in the first trimester are limited and remain relatively rare in the second trimester [38,39]. There are several logical reasons why the third trimester poses the highest risk of infection. The increased placental surface area and enhanced blood flow to the placenta during this stage provide more points of contact with potential pathogens, increasing the bacterial load that can reach the placenta. Additionally, as the placenta begins to thin to facilitate nutrient transfer to the growing fetus, it also becomes more permeable to pathogens [40]. Furthermore, the third trimester is characterized by heightened activity of the innate immune system, including neutrophils, macrophages, and the complement system. While these immune components help to fight infections, they also contribute to inflammatory conditions that may complicate pregnancy. This phase, necessary for labor initiation, coincides with a shift from Th2 (anti-inflammatory) to Th1 (pro-inflammatory) cytokine profiles, which primes the maternal body for childbirth but increases susceptibility to pathogens that thrive in an inflammatory environment [12,41]. The concentration of listeriosis cases during the third trimester is also evident in a retrospective study that spanned an impressive duration of eight years [42].

This vertical transmission can lead to neonatal listeriosis. Less frequently, a fetus may acquire the infection during delivery if exposed to bacteria present in the birth canal. Although neonatal listeriosis is rare, it is a potentially severe condition with a high mortality rate, ranging from 20 to 30% [9].

**Figure 6:**
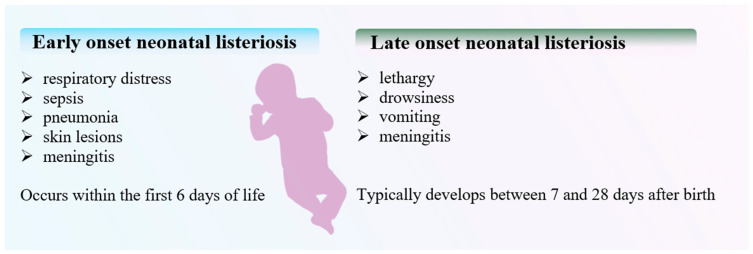
Early- and late-onset neonatal listeriosis, with its symptoms.

Neonatal listeriosis can manifest in two distinct forms (Figure 6). Early-onset disease, occurring within the first 6 days of life, is characterized by symptoms such as respiratory distress, sepsis, pneumonia, skin lesions, or meningitis. This form is particularly common among preterm infants. Late-onset disease typically develops between 7 and 28 days after birth, presenting with symptoms including lethargy, drowsiness, vomiting, or meningitis. Early-onset listeriosis is associated with a greater risk of severe complications and mortality [43]. A definitive diagnosis of neonatal listeriosis necessitates the isolation of LM from normally sterile sites, such as blood, cerebrospinal fluid, or amniotic fluid. Additionally, isolation may be achieved from oropharyngeal secretions, placental tissue, urine, or external sites like the conjunctiva, ear, nose, or throat [37]. A severe form of early-onset neonatal listeriosis is granulomatosis infantisepticum. This condition is characterized by the widespread formation of granulomas in various organs and tissues of the fetus or newborn, including the liver, spleen, lungs, kidneys, brain, and skin. Granulomatosis infantisepticum is associated with a high mortality rate, which can reach up to 80%, particularly among preterm infants. A hallmark of this condition is the presence of distinctive skin lesions, which may appear as nodules or papules that are red or blue-gray in color. These lesions are typically visible at birth or shortly thereafter. In addition to granulomatous inflammation, the disease often presents with sepsis and meningitis [44,45].

In addition to the well-documented relationship between gestational age and the timing of listeriosis infection, the timing of maternal antibiotic treatment is a crucial prognostic factor. Administering antibiotics to the mother at least one day prior to delivery has been significantly associated with a reduction in the severity of neonatal listeriosis. This proactive treatment is linked to a decreased need for interventions such as inotropic support, fluid resuscitation, and mechanical ventilation in affected newborns. Timely maternal antibiotic therapy has been demonstrated to reduce the likelihood of severe neonatal presentation by 77%. The effectiveness of this strategy relies on accurate and prompt diagnosis. The substantial benefits observed, including a notable reduction in the incidence of neonatal infection associated with early maternal treatment, suggest that preemptive antibiotic therapy may be justified in cases where maternal-fetal listeriosis is suspected, even before culture results are available [46,47].

The management of neonatal listeriosis primarily involves the prompt administration of antibiotics to prevent complications and reduce mortality. Ampicillin is the first-line treatment and is often combined with gentamicin to achieve a synergistic effect, particularly in severe cases such as those involving sepsis or meningitis. The typical duration of treatment ranges from 14 to 21 days for early-onset sepsis, with an extended duration required if complications like meningitis or brain abscesses are present. Penicillin G can serve as an alternative to ampicillin. Supportive care, including intravenous fluids and respiratory support, is frequently necessary for infants with severe symptoms. In cases where meningitis is present, antibiotics such as ceftriaxone or meropenem may be considered, depending on the clinical severity and local resistance patterns [9].

The prognosis of neonatal listeriosis is markedly influenced by the timing of diagnosis and the initiation of treatment. Early-onset listeriosis is associated with a higher mortality rate due to its correlation with preterm birth and severe sepsis; however, timely antibiotic therapy can enhance survival outcomes. Conversely, while late-onset form is less immediately fatal, it carries a risk of long-term neurological complications, particularly in cases involving meningitis. Survivors of neonatal listeriosis, especially those who have experienced meningitis, may encounter developmental delays, hearing loss, or other neurological impairments. These potential long-term effects highlight the critical need for rigorous follow-up and early intervention therapies in order to effectively address and manage the residual impacts of the disease [46].

## 6. Discussion

This article tries to present a comprehensive exploration of *Listeria monocytogenes* and its significant impact on pregnant women, neonates, and other high-risk populations. Through an in-depth analysis of Listeria’s biology, pathogenicity, and its role in causing listeriosis, the article highlights the critical need for awareness, prevention, and timely intervention.

*Listeria monocytogenes* is not just another foodborne pathogen but one that poses severe risks, especially to pregnant women. The bacterium’s unique ability to thrive under diverse and harsh environmental conditions, such as refrigeration, and its ability to form biofilms make it a persistent threat in food processing and storage environments. This adaptability, coupled with its intracellular lifestyle, underscores the challenges in controlling its spread and the importance of stringent food safety practices.

The susceptibility of pregnant women to listeriosis remains an underrated problem. Pregnancy alters the immune response, making women more vulnerable to infections like listeriosis. The bacterium’s affinity for the placenta and its ability to cross the placental barrier is of particular concern as it can lead to severe outcomes, including miscarriage, stillbirth, preterm labor, and neonatal listeriosis. The analysis of serotypes and clonal complexes associated with adverse pregnancy outcomes provides insights into the pathogen’s specific adaptations that increase its virulence in this context.

Diagnosing listeriosis in pregnant women remains challenging due to the nonspecific nature of symptoms and the bacterium’s ability to evade immune detection. We highlight the importance of targeted diagnostic techniques, such as placental cultures and PCR testing, which are critical for confirming listeriosis in pregnancy. Furthermore, an overview of treatment strategies, particularly the use of antibiotics like ampicillin and gentamicin, emphasizes the necessity of prompt intervention to mitigate the severe consequences of the infection.

From a public health perspective, more proactive measures should be taken to educate pregnant women about the risks of listeriosis and the importance of avoiding high-risk foods. The role of healthcare providers in recognizing and managing listeriosis, especially in high-risk populations, is also crucial for preventing adverse pregnancy outcomes.

Moving forward, further research into the specific virulence factors of *Listeria monocytogenes* and its interactions with the host immune system could provide new avenues for prevention and treatment. The development of more sensitive and rapid diagnostic tools, as well as vaccines, could significantly improve outcomes for pregnant women and their newborns. Additionally, public health policies should prioritize the control of Listeria in food production and enhance guidelines for pregnant women to prevent listeriosis.

## 7. Conclusions

While listeriosis may be rare, its impact, particularly in pregnancy, is profound. The insights provided in this article should encourage both healthcare providers and policymakers to continue to improve strategies for the prevention, early detection, and effective treatment of this often overlooked but dangerous pathogen.

## Figures and Tables

**Table 1:** Most common sources of *Listeria monocytogenes*.

Source	Commentary
Dairy products	LM can contaminate raw milk, potentially resulting in the contamination of dairy processing facilities and the production of dairy products derived from the contaminated milk.
Meat products	While raw meats can become contaminated, the risk is significantly higher with processed, ready-to-eat meats. Meat slicers used in retail settings have been identified as a key source of potential cross-contamination.
Poultry products	Poultry is more frequently contaminated with LM compared to beef, which is likely due to the increased exposure of chickens to contamination from other carcasses and processing equipment during production.
Vegetables	Vegetables can become contaminated with LM through contact with contaminated soil.
Animals	Numerous animals, including cattle, poultry, and sheep, can harbor and excrete LM asymptomatically. This asymptomatic carriage can lead to environmental contamination and, subsequently, the potential contamination of food products.
Household kitchens	Given the widespread presence of LM in food, household kitchens are at risk of contamination, especially in dishcloths and refrigerators, for example.
Nosocomial sources	Although rare, listeriosis can be transmitted within hospital environments, particularly in neonatal units.

**Table 2:** To reduce the risk of infection, certain foods should be avoided during pregnancy.

Food	Commentary
Ready-to-eat deli meats and hot dogs	Unless reheated to an internal temperature of 74 °C (165 °F) or until steaming hot, pregnant women are advised to avoid certain foods due to the risk of LM contamination.
Refrigerated or chilled smoked seafood	Products such as smoked salmon, trout, tuna, cod, whitefish, and mackerel pose a particular risk unless properly cooked to the recommended internal temperature 74 °C (165 °F) or steaming hot.
Refrigerated pâtés or meat spreads	These products are also best avoided during pregnancy due to the risk of LM contamination; however, canned or shelf-stable versions are generally considered safe for consumption.
Unpasteurized (raw) milk and foods containing it	This includes cheeses, ice cream, and other dairy products made with unpasteurized milk.
Soft cheeses	Cheeses such as feta, blue-veined varieties, brie, camembert, queso blanco, queso fresco, and panela carry a heightened risk of LM contamination, unless explicitly labeled as being made from pasteurized milk.
Unwashed raw produce	Fruits and vegetables should be thoroughly washed before consumption, regardless of whether they will be peeled or cut, in order to eliminate any potential LM contamination.

## Data Availability

No new data were created or analyzed in this study. Data sharing is not applicable to this article.
